# Coexisting flavonoids and administration route effect on pharmacokinetics of Puerarin in MCAO rats

**DOI:** 10.1515/biol-2020-0053

**Published:** 2020-07-03

**Authors:** Pengyue Li, Linying Zhong, Linjie Yang, Jie Bai, Yang Lu, Shouying Du

**Affiliations:** Department of TCM Pharmaceutics, School of Chinese Materia Medica, Beijing University of Chinese Medicine, Beijing, 100102, China

**Keywords:** Puerarin, intranasal, stroke, *Radix Puerariae* Extract, pharmacokinetic

## Abstract

A pharmacokinetic comparison was made to evaluate the influence from other components in the *Radix Puerariae* Extract on pharmacokinetic behavior of Puerarin. Samples of blood and brain were collected by microdialysis and determined by high-performance liquid chromatography–mass spectrometry (MS)/MS. Pharmacokinetic parameters were estimated from the concentration versus time data using non-compartmental methods. In addition, a comparative pharmacokinetic study of Puerarin in stroke rats was studied after administration of the *Radix Puerariae* Extract via different routes to find an effective way to deliver drug into brain. Obvious pharmacokinetic differences were also observed in comparison between the Puerarin group and the *Radix Puerariae* Extract group based on middle cerebral artery occlusion (MCAO) rats. The *C*_max_ and area under the curve (AUC) of Puerarin in olfactory bulb of the Extract group significantly reduced when it was intravenously administered. However, the AUCs of Puerarin in plasma are 134.72 and 1707.02 mg/L min, via intranasal and intravenous administration of the *Radix Puerariae* Extract, respectively. The AUC of the intranasal group in brain is seven times higher than that of intravenous administration. Other ingredients in the Extract may affect the disposition of Puerarin and its transportation through the blood–brain barrier via intravenous administration. But intranasal administration is an effective route to deliver isoflavone-C-glycoside with poor hydrophilicity into brain.

## 1 Introduction

*Radix Puerariae* (*Pueraria lobata* (Willd.) Ohwi) is a traditional Chinese herbal medicine, which has been described by many ancient herbal treatises, such as Shen–Nong–Ben–Cao–Jing (the first pharmaceutical monograph in the history of China) and Compendium of Materia Medica (an ancient encyclopedia of Traditional Chinese Medicine written by Li Shizhen in Ming Dynasty). Nowadays, it is also introduced in Chinese Pharmacopoeia, Japanese Pharmacopoeia Korea Pharmacopoeia and Hong Kong Chinese Materia Medica Standards. *Radix Puerariae* is widely used in East Asian countries and regions in clinic for its therapeutic effects on ischemic cerebrovascular disease. In China, it has been considered either as an herbal medicine or as a source of food. It can be found in many healthy products, even beverages on sale in China.

Preliminary studies showed that the key therapeutic components are flavonoids. It has good antioxidant activity both *in vitro* (1,1-diphenyl-2-picrylhydrazyl scavenging activity) and *in vivo* (reducing the level of malondialdehyde) and antidepressant effect on depressive mice induced by cerebral ischemia reperfusion.

The main constituent of total flavonoids from *Radix Puerariae* is Puerarin and its anti-inflammatory, antioxidant, anticoagulant and protection effects on neurons damaged by glutamic acid have been reported mainly during the past few decades [1]. Puerarin has now been developed into an intravenous injection solution in China. Besides Puerarin, there are many other flavonoids in the extract (total flavonoids), such as 3′-methoxypuerarin, 3′-hydroxypuerarin and daidzin. Studies showed that they all possess protective effects on the brain or estrogen-like activity [2]. They may produce a synergistic effect when coupled with Puerarin. So, patients can be treated by the *Radix Puerariae* Extract instead of Puerarin, just like the famous herbal medicine, *Ginkgo biloba* extract, which can simplify the purification process.

Reports showed various pharmacokinetics of Puerarin in the *Radix Puerariae* Extract [3], based on healthy animals. However, the pharmacokinetics in disease was seldom reported. Besides, though *Radix Puerariae* was studied for a long time, the pharmacokinetics of Puerarin has never been compared between the Extract group and the Puerarin group.

Therefore, the primary objective of this study was to investigate the effects of other components in total flavonoids on the pharmacokinetic behavior of Puerarin by comparing the parameters of the Extract group (total flavonoids) and the Puerarin group. For ischemic cerebrovascular disease, the intranasal drug delivery system has become the hotspot of drug development in recent years for its convenience and high brain target. So, in this study, the *Puerariae* extract was administered via intranasal route and intravenous route and the pharmacokinetic parameters were compared with each other.

## 2 Materials and methods

### 2.1 Chemicals

The standards of Puerarin and Naringin were purchased from National Institutes for Food and Drug Control (Beijing, China), and sodium chloride was obtained from Beijing Chemical Reagents Company (Beijing, China). For the mobile phase, methanol (CH_3_OH) was purchased from Mreda Technology Inc. (USA), and high-purity water was supplied by Hangzhou Wahaha Group Co., Ltd (Zhejiang, China). Puerarin (crude drug for Puerarin injection with a purity of 96.06%) used for administration was purchased from Ankang Zhengda Pharmaceutical Company (Shanxi, China).

### 2.2 *Radix Puerariae* Extract (total flavonoids)

*Radix Puerariae* total flavonoids were prepared and purified by researchers in our lab as follows. *Radix Puerariae* decoction was concentrated into 1.05–1.10 g/mL, and 95% ethanol was added to precipitate the impurities. After that, macroporous resin was used to purify the flavonoids [4]. The total flavonoids in the final product were determined by ultraviolet spectrophotometry to be about 72%. The Puerarin content was measured by high-performance liquid chromatography (HPLC). The Puerariae extract was ultrasonicated with water for 10 min. The solution was then filtered through the 0.22 µm microporous membrane before injection. The analysis was conducted with methanol–water by gradient elution at a flow rate of 1.0 mL/min and detected at the wavelength of 250 nm. The content of Puerarin in the Extract was about 35.06% [4]. There are five main ingredients in the extract (shown in the chromatograph at the wavelength of 250 nm), which were identified as 3′-hydroxypuerarin, puerarin, puerarin xyloside, 3′-methoxypuerarin and daidzin by ultra performance liquid chromatography (UPLC)-mass spectrometry (MS)/MS.

### 2.3 Preparation of intravenous injection and intranasal drop

Puerarin injection was used in clinic at a dosage of 200 mg/d for an adult. So, the dosage is about 5 mg for each rat with a weight of about 250 g. The structure of main ingredients in the extract is similar to Puerarin, and they are also effective in treating ischemic cerebrovascular disease. So, each rat was treated with a dosage of 5 mg extract (containing 35.06% Puerarin).

*Radix Puerariae* Extract and Puerarin dissolved in ethanol–propylene glycol–saline solution (30/30/40, v/v/v) to reach the concentration of 150 mg/mL (52.59 mg/mL for Puerarin) and 73 mg/mL (70.12 mg/mL for Puerarin) were named as total flavonoids nasal drop and Puerarin nasal drop, respectively.

Total flavonoids and Puerarin dissolved in saline to reach the concentration of 5 mg/mL (1.75 mg/mL for Puerarin) and 1.8 mg/mL (1.73 mg/mL for Puerarin) were called total flavonoids injection and Puerarin injection, respectively.

### 2.4 Animal stroke models

The Sprague-Dawley rats (SPF animal, male, 250 ± 20 g, 8-week-old) were purchased from Vital River Laboratory Animal Technology Co. Ltd (VRL, Beijing, China). All animals were housed in a room under standard conditions of temperature and light. Before the study, they were fasted for 12 h with free access to water.

Middle cerebral artery occlusion (MCAO) model was obtained according to the method of Longa et al. [5]. Briefly, following a midline incision in the neck of each rat, the right common carotid artery (CCA), the external carotid artery (ECA) and the internal carotid artery (ICA) were separated. After the ECA and the CCA were ligated, a 0.26 mm polylysine-coated nylon monofilament (supplied by Beijing Shadong Biotechnology Co., Ltd) was inserted into the MCA through ICA. The filament was slowly withdrawn after keeping the ischemic state for 2 h.


**Ethical approval:** The research related to animal use has been complied with all the relevant national regulations and institutional policies for the care and use of animals. All experimental procedures were conducted in compliance with The Guiding Principles for the Care and Use of Laboratory Animal (published by Ministry of Science and Technology of the People’s Republic of China, Chapters 2, 3 and 5) and were approved by the Institutional Animal Care and Use Committee of the Beijing University of Chinese Medicine.

### 2.5 Drug administration

There were five rats in each group. For the intravenous group, infusion was administered through the implanted drug deliverer at a volume of 1 mL for each rat, with a speed of 10 µL/min. For the intranasal group, the *Radix Puerariae* Extract nasal drop was administered through the left nostril with a volume of 33 µL for each rat, whereas the Puerarin nasal drop was 25 µL for each rat.

Drug administration was started immediately when the filament was withdrawn. Nasal drop was administered in 5 min and the administration of infusion would last for 100 min.

### 2.6 Sample collection and determination

Microdialysis was used to collect the samples. The brain probe was implanted into the left olfactory bulb according to The Rat Brain in Stereotaxic Coordinates and the brain section (anteroposterior: +8 mm, mediolateral: +1 mm, dorsoventral: −1 mm relative to the bregma, a stereotaxic frame was used to position the probes precisely every time). The blood probe was implanted into the right jugular vein.

For sampling, a solution of saline was pumped through the probe using a CMA 402 pump with a flow rate of 1.5 µL/min. After drug administration, dialysate in the dead volume was discarded. Then, samples were collected from the outflow tube of the probe at intervals of 20 min for 5 h. All samples were stored at 4°C before analysis.

Naringin was used as the internal standard. Before analysis, Naringin solution (800 ng/mL) of 30 µL was mixed with the dialysate.

The analysis was performed on an Accela HPLC system equipped with a TSQ Quantum Access tandem mass spectrometer (TSQ Quantum Access MAX; Thermo Fisher Scientific Inc., Boston, USA).

A C_18_ column (Agilent Eclipse Plus, 3.5 µm, 100 × 4.6 µm) was used at 30°C, eluted with CH_3_OH (A) and water (B) in a gradient manner at the flow rate of 1 mL/min. The elution was carried out as follows: 0–8.0 min (24% A), 8.0–15.0 min (24–60% A), 15.0–17.0 min (60–24% A) and 17.0–20.0 min (24% A). The mass spectrometer was operated in the negative ionization mode using selective reaction monitoring to detect Puerarin and Naringin. The collision energy was 25 eV for Puerarin and 30 eV for Naringin. The precursor-to-production transition for Puerarin was *m*/*z* 415 → 295, and for Naringin it was *m*/*z* 579 → 295. The spray voltage, sheath gas and capillary temperature were set to be 3,500 V, 10 psi and 300°C.

### 2.7 Statistical analysis

The drug concentrations in olfactory bulb and plasma were calculated according to the following equation:

(1) {\text{Concentration}}_{\text{(Olfactory}\text{bulb}\text{or}\text{plasma)}}=\hspace{1em}{\text{Concentration}}_{\text{(dialysate)}}/{\text{Recovery}}_{\text{(}invivo\text{)}}.

The *in vivo* recovery of probe was tested by the no-net-flux-method. Blood probe and brain probe were implanted in the jugular vein and olfactory bulb by surgery described above. Normal saline with different concentrations of Puerarin was used as a perfusate and dialysate samples were collected for each concentration. The concentration difference between perfusate and dialysate (*C*_perfusate_ − *C*_dialysate_) versus the difference between perfusate and tissues (*C*_perfusate_ − *C*_plasma or olfactory bulb_) was plotted. The slope was calculated by the least square method, which was considered as the *in vivo* recovery of the probe, as shown in the following equation:

(2) {\mathrm{C}}_{\text{perfusate}}-{\mathrm{C}}_{\text{dialysate}}=\hspace{1em}\text{Recovery}\times ({C}_{\text{perfusate}}-{C}_{\text{plasma}\text{or}\text{olfactory}\text{bulb}}).

The pharmacokinetic parameters were calculated by the non-compartmental method using Kinetica 4.4 (Innaphase, MA, USA). Statistical comparisons among groups were performed with SPSS Version 17.0 (SPSS Inc., Chicago, IL, USA). Differences were considered significant at *p* < 0.05.

## 3 Results

**Figure 1:**
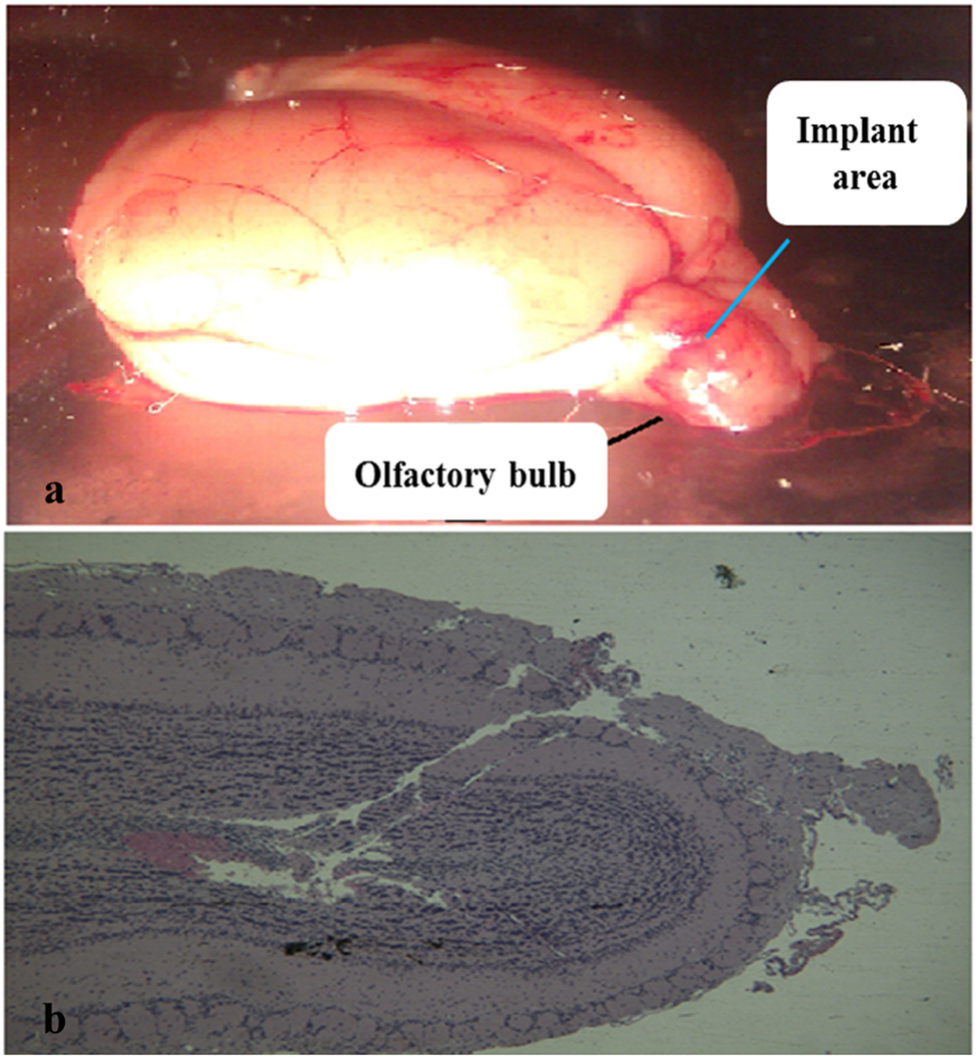
Olfactory bulb and its sagittal plane. (a) Brain of SD rat and (b) sagittal plane of olfactory bulb after implanting probe.

The HPLC-MS/MS method of Puerarin in plasma and brain in the present study was specific and efficient. The methodology of this research and the recovery of the probe were qualified as reported before. There was a good linearity between concentration (*C*) and the peak area ratio of Puerarin to internal standard; *C* = 0.23816 ratio − 0.0001 (for the concentration range of 0.002–0.111 µg/mL, *r* = 0.998) and *C* = 0.25562 ratio − 0.01582 (for the concentration range of 0.111–8.9 µg/mL, *r* = 0.999). The smallest concentration (0.002 µg/mL) of the calibration curve was taken as limit of quantitation. The precision of low, middle and high concentration is 6.11%, 4.86% and 2.61%, respectively. The *in vivo* recovery of probe was tested by the no-net-flux-method (*n* = 3, three probes were tested, respectively, for blood probe and brain probe). The recovery of the blood probe and that of brain probe were 17.52 ± 2.80% and 29.13 ± 1.72% [6], as shown in Table 1. Olfactory bulb and its sagittal plane are shown in Figure 1.

**Table 1:** Recovery of blood probe and brain probe (*n* = 3)

	Slope of regression line	
	Blood probe (%)	Brain probe (%)
1	18.84	27.14
2	14.31	30.10
3	19.42	30.15
Average ± SD	17.52 ± 2.80	29.13 ± 1.72

### 3.1 Pharmacokinetics comparison of Puerarin between Puerarin and *Radix Puerariae* Extract groups via intravenous administration route

The main ingredients in the extract are flavonoids, and they may interact with each other in disposition and transportation through blood–brain barrier (BBB). So, pharmacokinetic parameters of the Extract group and the Puerarin group [7] were calculated by the non-compartmental method and compared with each other.

**Figure 2:**
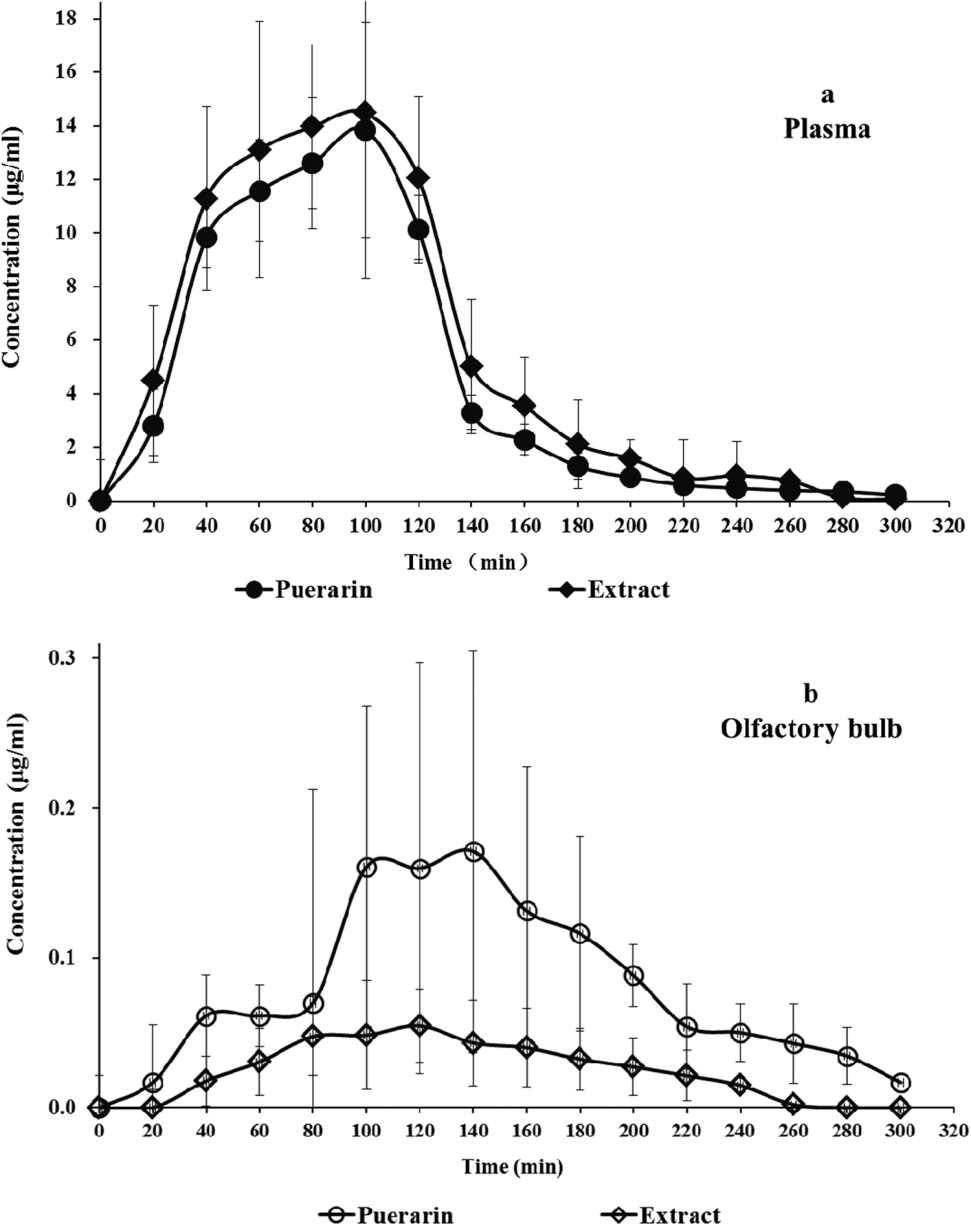
Plasma and olfactory bulb concentration–time curves of Puerarin after MCAO rats were treated with the *Radix Puerariae* Extract and Puerarin intravenously. (a) Concentration—time curves of plasma, (b) Concentration—time curves of olfactory bulb.

For i.v. administration, the plasma concentration curves of both the Puerarin group and the Extract group represented similar tendency (Figure 2), and the *C*_max_ and area under the curve (AUC) of the two groups were nearly the same. But the average value of *t*_1/2_ of the Extract group is much smaller than that of the Puerarin group.

In olfactory bulb, different behaviors were observed. The *C*_max_ and AUC of the Extract group were much smaller than that of the Puerarin group. However, *t*_1/2_ of the Extract group was longer than that of the Puerarin group. The parameters are shown in Table 2.

**Table 2:** Pharmacokinetic analysis of Puerarin after administration of the *Radix Puerariae* Extract and Puerarin intravenously based on the MCAO model (*n* = 5, in each group)

Parameters	Plasma		Olfactory bulb	
	Puerarin group	Extract group	Puerarin group	Extract group
*C*_max_ (µg/mL)	13.84 ± 2.45	14.96 ± 3.55	0.19 ± 0.12	0.06 ± 0.03*
*T*_max_ (min)	100 ± 0	104 ± 8	104 ± 38	108 ± 16
AUC_0–5h_ (min·µg/mL)	1388.62 ± 222.47	1707.02 ± 409.54	24.56 ± 15.50	7.39 ± 4.16*
*t*_1/2_ (min)	67.57 ± 39.65	31.23 ± 3.27	41.63 ± 14.19	73.49 ± 26.24*
CL (mL/min)	1.27 ± 0.25	1.07 ± 0.26	—	—

*C*_max_: peak concentration; *T*_max_: peak time; AUC: area under the curve; *t*_1/2_: half-life period; CL: clearance.

* *p* < 0.05 vs Puerarin group.

### 3.2 Pharmacokinetics of Puerarin in MCAO rat plasma and brain of extract group via different administration routes

**Figure 3:**
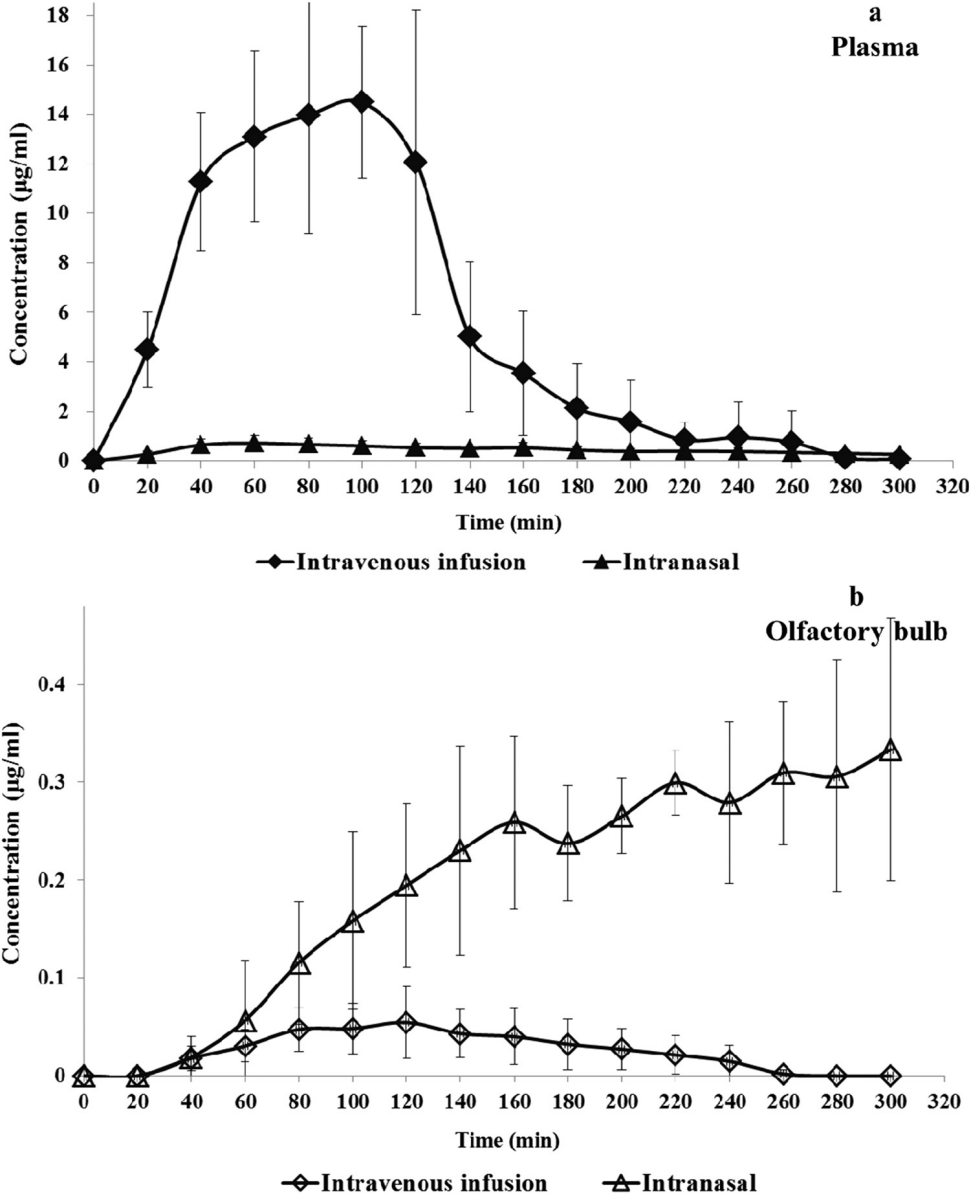
Plasma and olfactory bulb concentration–time curves of Puerarin after MCAO rats were treated with the *Radix Puerariae* Extract intravenously and intranasally. (a) Concentration—time curves of plasma, (b) Concentration—time curves of olfactory bulb.

The curves of Puerarin in stroke rats administered the *Radix Puerariae* Extract via intranasal and intravenous routes are plotted in Figure 3.

For intranasal administration, the Puerarin in plasma reached *C*_max_ at about 60 min after administration, and the *C*_max_ is 0.75 ± 0.27 µg/mL. After that, the concentration slowly reduced and produced a lower AUC in plasma.

For intravenous administration, the Puerarin concentration in plasma continuously increased in the first 100 min and the *C*_max_ is about 14.96 ± 3.55 µg/mL and the concentration rapidly declined later. But the AUC is 12 times higher than that of the intranasal administration group.

In the brain, the intranasal group completely demonstrated different pharmacokinetic behaviors. The Puerarin concentration in brain has a sustained growth in the sampling time, while the intravenous group reached *C*_max_ at 120 min and declined slowly after that. Obviously, the brain targeting of Puerarin was highly improved after the extract was administered via intranasal route. The parameters of the two groups are shown in Table 3.

**Table 3:** Pharmacokinetic analysis of Puerarin after administration of the *Radix Puerariae* Extract intravenously and intranasally based on the MCAO model (*n* = 5, in each group)

Parameters	Plasma		Olfactory bulb	
	i.v. infusion	i.n.	i.v. infusion	i.n.
*C*_max_ (µg/mL)	14.96 ± 3.55	0.75 ± 0.27*	0.06 ± 0.03	0.37 ± 0.10*
*T*_max_ (min)	104 ± 8	68 ± 16	108 ± 16	248 ± 53
AUC_0–5h_ (min µg/mL)	1707.02 ± 409.54	134.72 ± 33.64*	7.39 ± 4.16	58.60 ± 12.95*
*t*_1/2_ (min)	31.23 ± 3.27	140.16 ± 20.87*	73.49 ± 26.24	—
DTI	—	—	0.43%	43.50%

i.v.: intravenous; i.n.: intranasal; *C*_max_: peak concentration; *T*_max_: peak time; AUC: area under the curve; *t*_1/2_: half-life period; DTI: drug target index.

* *p* < 0.05 vs i.v. infusion group.

## 4 Discussion

### 4.1 *Radix Puerariae* Extract

*Radix Puerariae* Extract was prepared and purified in our lab as reported before. The contents of Puerarin and total flavonoids in the extract were tested by an HPLC and a UV-Vis Spectrophotometer, respectively. The contents of Puerarin and flavonoids in the extract were greatly improved after the purification procedure, by up to 35 and 72%, respectively. UPLC-MS/MS was used to analyze the extract and 3′-methoxypuerarin, 3′-hydroxypuerarin, daidzin and puerarin-*O*-xyloside are the main ingredients in the extract.

### 4.2 Pharmacokinetic comparison between Puerarin and Extract groups

There are many components in effective fractions of herbal medicines, which are considered as the basis of multi-target and multi-function in the treatment of diseases. However, the interactions between the ingredients are still unknown. Only a few studies were carried out to investigate it. A study in China showed that other ingredients in the ether fractions of Salvia could improve the absorption of Cryptotanshinone [8]. But Liu et al. [9] found the opposite result in the study of total flavonoids from epimedium. In this study, we compared the pharmacokinetic behaviors of Puerarin in MCAO rats treated with Puerarin and total flavonoids from *Radix Puerariae* via intravenous route and significant differences were observed. Compared with the non-compartment model, the compartment model is more accurate toward analysis of dynamic behavior, which may provide more information on the effect on Puerarin disposition from other flavonoids. But, compartmental analysis is sensitive to interindividual variation. So, we still used the non-compartment model in this comparison.

Although there was no difference between the two groups on AUC_blood_ and *C*_max_, the average value of *t*_1/2_ of the Puerarin group was longer than the Extract group (the SD value of the Puerarin group was larger, but no outlier was observed by the Grubbs test and the Dixon test), which may indicate that other flavonoids in the extract may slightly accelerate the disposition of the Puerarin. A bold guesswork is that other flavonoids in the extract may induce the expression of enzymes in phase I and II reactions in hepar. Guerra et al. [10] investigated the effect of the Pueraria extract on different cytochrome P450 (CYP) isoforms and found that the *Radix Puerariae* Extract could increase testosterone 6β-, 16α- and 16β-associated hydroxylases, which may relate to the metabolism of Puerarin. However, more experiments still need to be done to verify it, especially experiments based on liver microsomal incubation systems.

In olfactory bulb, the *C*_max_ and AUC of the Extract group are about 1/3 of the Puerarin group. It was supposed that there was a competition (or a trend of competition) between these isoflavone-*C*-glycosides when they pass through the BBB. The BBB is a complex and highly selective barrier interposed between the blood and central nervous system (CNS). Due to the “tight junctions” and permselectivity, for most of the drugs, it is a challenge to pass through the BBB. Liang et al. [11] studied the transport properties of Puerarin in the Pueraria extract in the Caco-2 cell model, and the results suggested that both passive diffusion and active transportation occurred in the process of absorption. The permeability of Puerarin could be affected by the P-gp and MRP inhibitors such as Cyclosporin A and MK-571. We studied the ingredients in the Pueraria extract by an UPLC-MS-MS, according to the molecular weight information, and there are five main ingredients in it as follows: 3′-hydroxy puerarin, puerarin, puerarin xyloside, 3′-methoxy puerarin and daidzin. They all have similar structure, and they may compete with each other when they transport through the BBB. Interestingly, in contrast with plasma, *t*_1/2_ of the Extract group in olfactory bulb is longer than that of the Puerarin group. Although Puerarin is an isoflavone-C-glycoside with poor hydrophilicity, but its log *K*_o/w_ is −0.35, which indicates that the solubility of Puerarin in lipid is even worse than in water. However, brain is a tissue with abundant phospholipid and the other ingredients in the extract may compete with Puerarin and decrease its disposition in brain. So, it will take a longer time for the brain to metabolize Puerarin in the Extract group, compared with the Puerarin group.

We did not compare the parameters of the two groups when drugs were administered intranasally as it is difficult to draw conclusions or make inferences. The enzymes and the mucociliary clearance in nose have a great influence on the process of absorption. Besides, the mechanism between stroke and olfactory nerve at the acute stage is still unknown. So, inference drawn from comparison between intranasal groups is meaningless.

### 4.3 Pharmacokinetic comparison between different administration routes

Most of the pharmacokinetic studies reported were based on healthy animals. But, the pathological state may bring changes to the pharmacokinetic behavior as reported before [12]. Previous study of Puerarin also showed that the *C*_max_ and AUC of olfactory bulb of MCAO rats are three times as high as those of healthy rats, after intranasal administration of puerarin. Besides, studies based on pathological animals may provide more valuable information to clinic. Therefore, in the study of *Radix Puerariae* Extract, we conducted the pharmacokinetic experiments in MCAO rats.

The AUC and brain targeting of Puerarin in the extract were highly improved when it was administered intranasally. Intranasal administration is a potential route to deliver isoflavone-*C*-glycoside with poor hydrophilicity into brain, as was shown in healthy rats [6]. It has been reported that puerarin could attenuate glutamate-induced cell injury in a dose-dependent manner and it can reduce the infarct volume dose-dependently. So, the drug dosage can be reduced after further investigations on pharmacodynamic tests. In addition, drug accumulation was observed in olfactory bulb in the intranasal group. Five MCAO rats were intranasally treated with the *Radix Puerariae* Extract and an accumulation of Puerarin in olfactory bulb happened in two of them (the drug concentration reached *C*_max_ at 300 min). The *T*_max_ values of the other three rats were 160, 220 and 260 min. So, it has a potential risk of toxicity caused by drug accumulation. Olfactory bulb is a tissue of great importance and studies found that it has connections with many encephalopathy [13], such as depression, Alzheimer’s disease, Parkinson’s disease, multiple sclerosis and stroke, although the mechanism has not been sufficiently uncovered. Injury of olfactory bulb may also bring CNS diseases. So, it is important to evaluate the accumulative toxicity of these flavonoids before intranasal delivery system developed. More time points should be added to monitor the concentration of Puerarin after 300 min. Besides, toxicology test should be carried out in different animals.

In our study, to get nasal drops with high concentration, Puerarin or Extract was dissolved in ethanol–propylene glycol–saline solution (30/30/40, v/v/v), which may irritate or even damage the nasal mucosal [14]. It is believed that new dosage forms such as liposomes, microemulsions and microspheres with low irritating effect can be prepared as drug carriers, which can also be transported into brain from nose efficiently [15].

Ischemic cerebrovascular disease is one of the most dangerous pathophysiologic events in the world with high mortality and morbidity. It is believed that the survival rate can be improved by emergency treatment. As injections can only be administered by specialists, it is inconvenient for most people to deal with a medical emergency for the difficult operation of injection administration. To some extent, this inconvenience may delay the best rescue time and increase the risk of physical disability or death, especially for the areas with insufficient health care resources. In China, stroke is the leading killer disease in suburban areas. Compared with injection, the intranasal drug delivery system is a convenient way with high brain targeting. Besides, it took only a few minutes for the drug to reach the brain, just a bit slower than intravenous administration, so it can come into effect soon after administration too. Up to now, there are no intranasal preparations for stroke on the market. We believe that a precise drug delivery device is the key factor to the launch of intranasal products for the emergency of brain diseases. In addition, drug combination is another way to improve the drug distribution in brain. In the history of China, herb medicines were not used separately, instead they were used in the form of formulas. Studies showed that volatile oils of some herb medicines can make a reversible opening of the BBB [16,17]. More drug can be delivered into brain if the Puerarin or *Radix Puerariae* Extract was combined with volatile oils. Moreover, application of bioadhesive dosage forms (like in situ gel delivery system and bioadhesive delivery system) [18,19] is helpful for the reduction of mucociliary clearance and prolonging of the drug residence time in the nasal cavity [20], which may also improve the drug distribution in brain.

## 5 Conclusion

This study focused on the ingredient interactions in the extract and showed that the other ingredients in the extract may affect the disposition of Puerarin and its transportation through the BBB. But more experiments need to be done to confirm it. It is necessary to make a comparison between Puerarin and total flavonoids through some pharmacodynamic experiments *in vivo*. If the effect of total flavonoid extract is equivalent to Puerarin or higher than that of Puerarin, the decline of AUC we observed above is acceptable. Comparative pharmacokinetics of intravenous and intranasal administration indicated that intranasal administration has obvious potential advantages in the treatment of brain disease for its high brain targeting under the pathology conditions and it may overcome the negative effects caused by other components in the extract.
